# A Novel Handwritten Digit Classification System Based on Convolutional Neural Network Approach

**DOI:** 10.3390/s21186273

**Published:** 2021-09-18

**Authors:** Ali Abdullah Yahya, Jieqing Tan, Min Hu

**Affiliations:** 1 School of Computer and Information, Anqing Normal University, Anqing 246011, China; 2 School of Computer and Information, Hefei University of Technology, Hefei 230009, China; jieqingtan@hfut.edu.cn (J.T.); jsjxhumin@hfut.edu.cn (M.H.)

**Keywords:** data augmentation, Root Mean Square Propagation (RMSprop), batch normalization, MNIST handwritten digit database, receptive field

## Abstract

An enormous number of CNN classification algorithms have been proposed in the literature. Nevertheless, in these algorithms, appropriate filter size selection, data preparation, limitations in datasets, and noise have not been taken into consideration. As a consequence, most of the algorithms have failed to make a noticeable improvement in classification accuracy. To address the shortcomings of these algorithms, our paper presents the following contributions: Firstly, after taking the domain knowledge into consideration, the size of the effective receptive field (ERF) is calculated. Calculating the size of the ERF helps us to select a typical filter size which leads to enhancing the classification accuracy of our CNN. Secondly, unnecessary data leads to misleading results and this, in turn, negatively affects classification accuracy. To guarantee the dataset is free from any redundant or irrelevant variables to the target variable, data preparation is applied before implementing the data classification mission. Thirdly, to decrease the errors of training and validation, and avoid the limitation of datasets, data augmentation has been proposed. Fourthly, to simulate the real-world natural influences that can affect image quality, we propose to add an additive white Gaussian noise with σ = 0.5 to the MNIST dataset. As a result, our CNN algorithm achieves state-of-the-art results in handwritten digit recognition, with a recognition accuracy of 99.98%, and 99.40% with 50% noise.

## 1. Introduction

In recent decades, the image classification problem has been widely addressed in the literature, and it is still an active research field in image processing today. In this field, convolutional neural networks have made a substantial breakthrough in visual recognition, especially handwritten digit recognition [1,2,3,4,5]. These networks have a great ability for learning and extracting image features easily. CNN architectures for image classification have two different types of layers: convolutional layers for extracting image features and fully connected layers for performing the classification task based on the features extracted by the preceding convolutional layers [6,7]. The handwritten digit recognition problem is a topic of heated debate in recent years. Despite that there are enormous convolutional neural network algorithms proposed for handwritten digit recognition, issues such as recognition accuracy and computation time still require further improvement [8]. In the literature, there are massive studies based on different techniques that have been proposed for handwritten digit recognition: in [9], Ali et al. have used MNIST handwritten digits as a dataset. The authors proposed to utilize DL4J framework for handwritten digit recognition and convolutional neural network as a classifier, and they achieved a reasonable accuracy of 99.21%.

Schaetti et al. proposed to utilize the reservoir computing paradigm to classify MNIST handwritten digit images, and they achieved an accuracy of 93%. The authors claim that their model outstrips other models such as simple feedforward networks with regard to error rate and complexity [10].

Hermans et al. [11] have addressed the MNIST handwritten digit classification problem. In this context, 10 iterations are used for each image in the MNIST dataset; in other words, each input digit is repeated for 10 masking periods. In their experiments, the authors focused on both an MNIST handwritten digit classification dataset, and a TIMIT phoneme classification dataset. In both MNIST and TIMT datasets, the authors found that optimizing the input encoding can make great improvements over random masks.

Mohapatra et al. [12] proposed a new method for classifying MNIST handwritten digit images. In their new method, the authors used the discrete cosine space-frequency transform to extract image features and artificial neural network classifiers to solve the classification problem. In order to reduce the computational cost, the authors proposed to normalize all the images of the MNIST handwritten digit dataset and exclude undesirable boundary pixels.

Kussul and Baidyk [13] proposed a new neural classifier limited receptive area (LIRA) for MNIST handwritten digit images classification. In the LIRA classifier, the sensor layer is followed with the associative layer, and the trainable connections are used to connect the associative layer with the output layer. Experiments with MNIST handwritten digit images show that the LIRA classifier has achieved a classification accuracy of 99.41%. In order to classify MNIST handwritten digit images, Ahlawata and Choudharyb [14] proposed to build a hybrid classification model by integrating convolutional neural networks and support vector machines (SVM). In this context, the authors used convolutional neural networks to extract the features of the image, while SVM was used as a binary classifier. Based on experimental results the authors have achieved a classification accuracy of 99.28%.

Chazal et al. [15] proposed to use identical network topologies to compare between two weight optimization methods using MNIST handwritten digit classification database. In the first weight optimization methods, the authors use the extreme learning machine algorithm. While backpropagation algorithm is used in the second weight optimization methods. Based on their experimental results, the authors found that the weight optimization method that uses the extreme learning machine is much faster than the one that uses the backpropagation algorithm.

In [16], Ma and Zhang adopted deep analysis with multi-feature extraction to build a handwritten digit classification method. In order to exclude negative information and maintain relevant features, the images of various sizes were normalized, and projection features were extracted from pre-processed images. Distribution features and projection features are also used to classify MNIST handwritten digit datasets. In [17], Lopes et al. addressed the MNIST handwritten digit classification problem and used the classifier optimum-path forest to classify this dataset. The authors used the signature of the characters as a feature extractor and the optimum-path forest algorithm as a handwritten digits classifier. According to the experimental results, the authors achieved an average accuracy of 99.53%.

Aly and Allotairi [18] proposed a new unsupervised deep convolutional self-organizing maps network where an MNIST handwritten digit database is used to evaluate the proposed network. The authors used 2D self-organizing maps to extract the features of the image. In their proposed network, the authors partitioned the input image (28 × 28) into small patches and represented each small patch by N-coordinates of the winner neuron in the deep convolutional self-organizing maps network. The authors have achieved remarkable results on the MNIST handwritten digits dataset. Nevertheless, they failed to achieve good results with the data augmentation technique. Man et al. [19] proposed to apply a single-hidden layer feedforward network to classify MNIST and USPS handwritten digit images. In this context, the authors adopted the batch learning type of least-squares to improve the input and output weights. In order to minimize the sensitivity of the single-hidden layer feedforward network, the authors proposed to adjust the regularization parameters. Supervised learning in spiking neural networks was adopted in [20] for handling the MNIST handwritten digit recognition problem. In this network, the authors have encoded all data and processed this data in the spike domain at sparse biological spike rates. In this sense, the authors claimed that utilizing the precise spike time for classification resulted in better classification accuracy. Based on the experimental results, the authors achieved a classification accuracy of 98.17%. To correctly represent the data in a feature space, Cardoso and Wichert [21] proposed to use the output of a biologically inspired model for visual recognition as a feature space. In this aspect, the output of a biologically inspired model is used to train the linear classifier, and MNIST and USPS handwritten digits datasets are used to evaluate the proposed model. In the training process, the authors proposed to reiterate the unsupervised learning 10 times and retain the best by cross-validation on the training set to select the stimuli for simple cell layers. Niu and Suen [22] proposed to combine two different artificial neural network classifiers, convolutional neural network and support vector machine. The authors used convolutional neural network as feature extractor to extract features from the raw images and used support vector machine as a classifier to classify the MNIST handwritten digit database. The authors achieved a recognition rate of 94.40%. However, the complexity of this hybrid model makes it impractical for some neural network applications such as self-driving cars.

Based on and support vector machine, Lauer et al. [23] proposed a new handwritten digits recognition method. In this method, the LeNet5 convolutional neural network is used as a feature extractor, and support vector machine is used as a classifier. In this aspect, the authors used affine transformations and elastic distortions to improve classification accuracy. However, the combination of these two networks made this method quite complex, which contributed to slowing the network speed.

In the literature, there is a large amount of CNN classification algorithms. However, most of these algorithms have not taken the following points into consideration: (1) appropriate filter size selection, (2) data preparation, (3) limitations in datasets, and (4) noise. For these reasons, they have failed to achieve remarkable enhancements in the image classification accuracy.

To address these drawbacks, our paper presents the following contributions: (1) After taking the domain knowledge into consideration, the size of the ERF is calculated. Calculating the size of the ERF plays an important role in selecting a typical filter size, and this, in turn, leads to improving the classification accuracy of our proposed CNN algorithm. In ERF, the process is started with a proposed filter to convolve the input image and gain its feature map. Then, this process is repeated with the next layers to gain deeper feature maps until getting the output image with an effective receptive field with a size of 22 × 22. The size (22 × 22) of the effective receptive field covers the entire image. (2) Unnecessary data leads to misleading results, and this, in turn, will negatively affect classification accuracy. To guarantee the dataset is free from any redundant or irrelevant variables to the target variable, data preparation is applied before implementing the data classification mission. Without applying pre-processing to the raw data, it is highly possible that unnecessary data will lead to misleading results. (3) In order to decrease the errors of training and validation as well as avoid the limitation of datasets, a data augmentation technique has been proposed. (4) To simulate the real-world natural influences that can affect image quality, adding additive white Gaussian noise with σ=0.5 to the MNIST dataset has been proposed.

In the proposed CNN architecture, the convolution layers, max-pooling layers, and batch normalization layers execute the feature extraction mission. Then, the feature maps learned from these layers are collected to classify the score of the image in the input layer.

Due to the limiting reliable training data, a data augmentation technique is the perfect solution for overfitting problems and generalization errors. In this work, we address the effects of the deficiency of training data on the recognition accuracy. Moreover, we apply the data augmentation technique to extract more information from the original dataset (MNIST dataset) to compensate for the lack of training data, which in turn leads to avoiding overfitting problems [24]. In this sense, different data augmentation techniques such as random rotation, zoom range, and random horizontal and vertical shifts are used in our experiments.

To guarantee that the dataset is free from any unnecessary details and is suitable to apply in our CNN model, data preparation is conducted as an essential first step in our proposed model.

## 2. Data Preparation

Data preparation can be considered as an initial step in machine learning classification models.

In some cases, statistical errors and noise in the dataset should be corrected before applying CNN.

Data that contains unnecessary details needs cleaning and formatting. To ensure that data was free from null, missing values, and other unnecessary data, and before implementing CNN to classify MNIST handwritten digits, we had to complete data preparation as an essential first step to guarantee this dataset was fit to use in CNN. Without applying pre-processing to the raw data, it is highly possible that unnecessary data leads to misleading results, and this, in turn, will negatively affect the classification accuracy.

In this work, the data preparation process involves the following steps:

### 2.1. Feature Density

**Figure 1:**
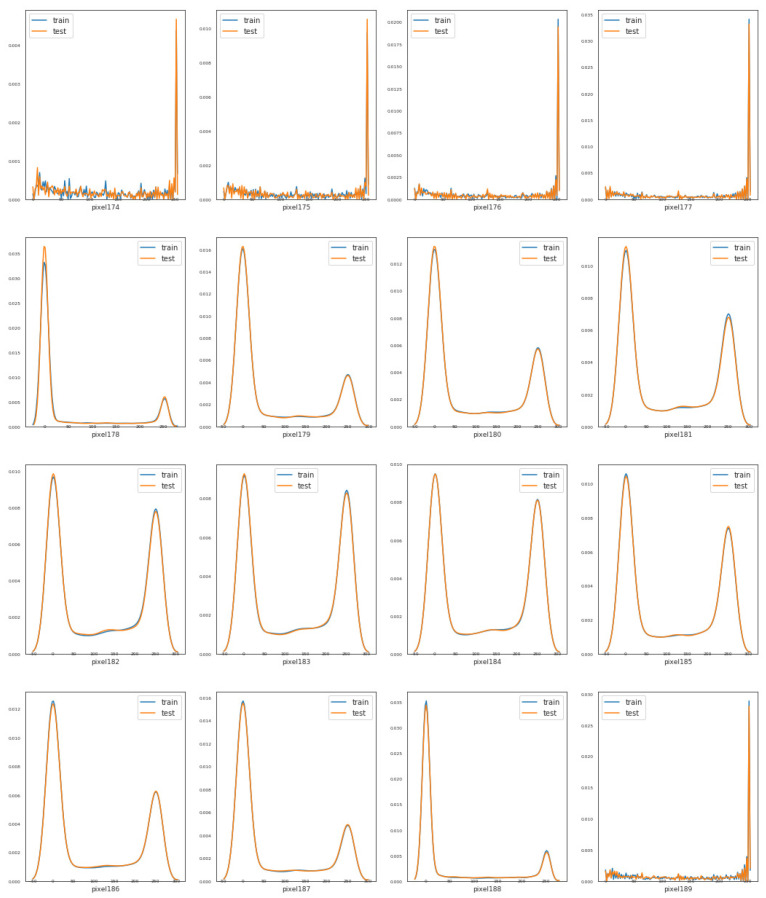
Density plot of features in training and testing dataset.

Our training and testing sets contain 784 numerical features, named from pixel 0 to pixel 783. For simplicity, we address the distribution of 16 features. From Figure 1, we can notice that features like pixel 174, pixel 179, pixel 185, and pixel 188 have different distributions, while some features such as pixel 174, pixel 181, and pixel 186 seem to have distributions that are similar to a bivariate distribution. Nevertheless, in general terms, the training and testing data seem to be well-balanced with respect to the distribution of the numeric features.

### 2.2. Mean and Standard Deviation of Training and Testing Sets

**Figure 2:**
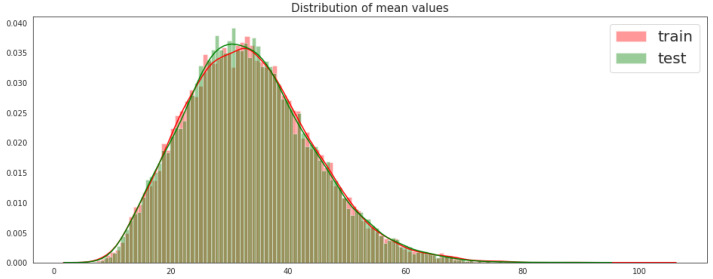
Distribution of the mean values of the training and testing sets.

From Figure 2, we can observe that the values of training and testing sets are quite identical, which indicates that the means of the features in training and testing data are very close. In this figure, most of the values cluster around the central peak, which means that the values in training and testing sets are normally distributed.

**Figure 3:**
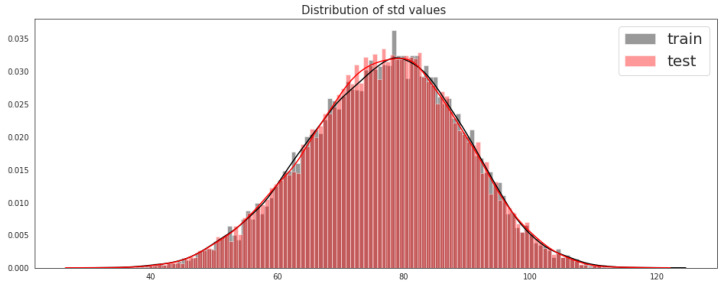
Distribution of the standard deviation of the training and testing sets.

From Figure 3, we can see that the training and testing data distribution tends to be close to the mean (clustered around the mean), which means that the standard deviation of this data is close to zero.

### 2.3. Mean Values of Labels in Training Set

**Figure 4:**
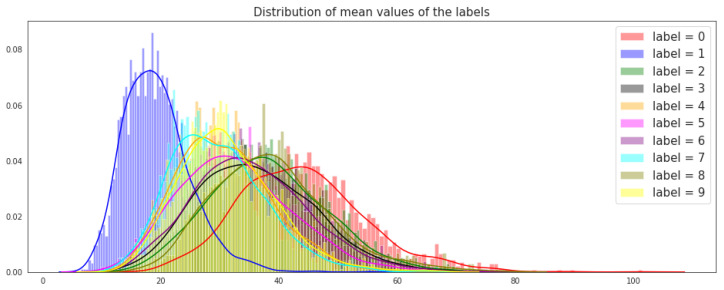
Distribution of the mean values of the labels in the training set.

**Figure 5:**
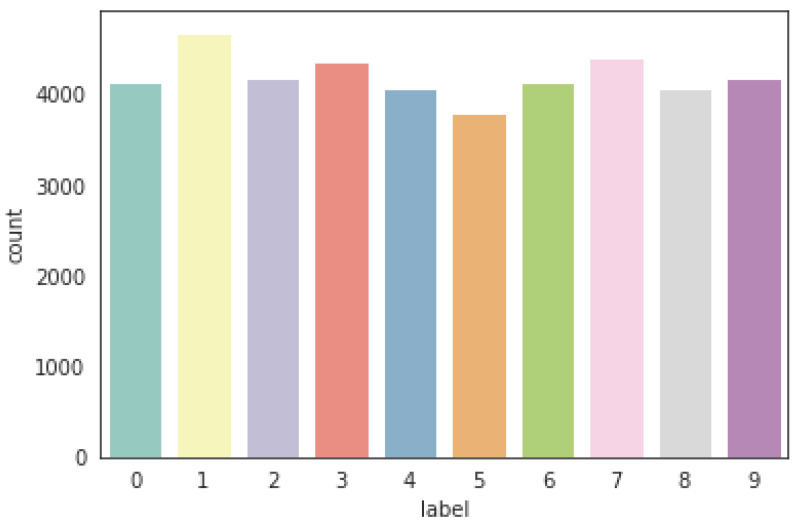
Distribution of target values in the training set.

From Figure 4, we can observe that the ten labels (0 to 9) have different averages, and this is logical because the different labels have different features. As shown in Figure 5, the different averages are consistent with the distribution of these labels.

### 2.4. Distribution of Label Values in Training Set

As shown in Table 1 and Figure 5, the 10 labels (digits) have almost the same counts, which means that the data is balanced with respect to the label values. These balanced labels will contribute to reducing the labels’ over-representation in the validation set during the random splitting of the training set.

### 2.5. Materials

The Modified National Institute of Standards and Technology (MNIST) dataset can be considered as one of the most common datasets, which contains binary images of handwritten digits. MNIST dataset consists of 70,000 grayscale images of size 28 × 28. In our experiments, we divide the 70,000 grayscale images into 420,000 samples (images) for training and 280,000 for testing. In the training and testing sets, there are 784 features.

## 3. Proposed CNN Architecture

**Figure 6:**
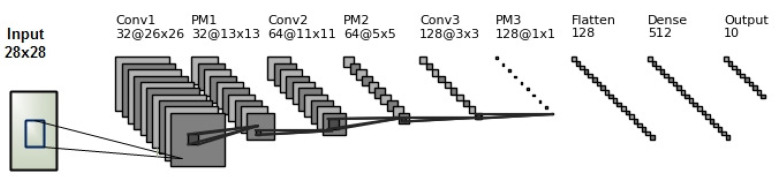
The architecture of our Convolutional Neural Network (CNN).

As shown in Figure 6, our convolutional neural network consists of an input layer, three hidden layers and a fully connected layer (classification layer). Each hidden layer contains: convolutional layer with rectified linear unit (ReLU), batch normalization layer, max pooling layers, and dropout layers. Classification layer includes: flatten layer, two dense (fully connected) layers, batch normalization, and dropout layers. In this paper, we use the flatten layer to convert the final feature maps into a one-dimensional vector and combine the local features of the preceding convolutional layers.

### 3.1. Input Layer

Our input image is a handwritten digit. This digit is in grayscale with a pixel size of 28×28×1 (width × height × channel). The range of pixel intensity values is from 0 (black pixel) to 255 (white pixel), and each value between these two numbers represents a different brightness level of the pixel.

### 3.2. Hidden Layer

Our hidden layer is a series of: convolution, rectified linear unit (ReLU), batch normalization, max pooling, dropout layers, and fully connected layers. In these layers, the features of the handwritten digits are extracted.

### 3.3. Convolution Layer

Convolution layer is used for extracting features from the input layer, where each N×N input neuron (in the input layer) is convoluted with f×f kernel, and in return delivery (N−f+1, N−f+1) as an output neuron.

In the convolution layer, output feature maps are produced by applying the convolution operations to the localized areas of the input feature maps.

The convolution layer contains a set of learnable kernels (filters) with limit dimensions [4]. During the convolution operations, kernels learn the features of the handwritten digits and produce more accurate classification.

Each kernel is applied on a subset of the input features that has the same size as the kernel. As shown in (1), each feature is multiplied by the corresponding value in the kernel. The multiplication operation result is called weight. After that, we add the bias, then apply a non-linear function (ReLU). This process is repeated until obtaining the output features map.

(1) y=σ(Wx+b),

where σ is the activation function (ReLU), *W* is the weight, *x* denotes the input features, and *b* is the bias. In this work, all fully connected and convolutional layers are covered by this formulation.

Because a handwritten digit database is nonlinear, introducing nonlinearity in CNN is highly needed. For that reason, a non-linear activation function (ReLU) is applied after each convolution operation. Applying ReLU after a convolution operation contributes to improving the CNN performance.

Since weights are determined by the kernel size, each neuron has a weight and bias the same as the kernel dimension that is connected to its receptive field. Usually, all neurons in the hidden layers have the same weights and biases. Consequently, the same feature in various parts of the input layer is detected by these neurons.

As is well known, the map that draws from the input layer to the hidden layer is known as a feature map.

In our proposed CNN architecture, we set 3×3 as the size of the filters in all convolutional layers.

As shown in Figure 6, our CNN has three convolution layers. $Conv2D_{1}$, $Conv2D_{2}$, and $Conv2D_{3}$, with 32, 64, and 128 filters of size 3×3×1 are used to generate 32, 64, and 128 feature maps, then rectified linear units (ReLUs, max(0,x)) are utilized for nonlinearity.

In our convolution operation, weights are shared throughout the image, and when kernels move throughout the image, we can easily extract the local features and detect the learned local patterns.

### 3.4. Rectified Linear Unit

Since the output of the convolution layer is considered as a linear operation, a nonlinear function that represents the biological neuron conduct is extremely required to be applied after the convolution operation.

Tanh ($f\left(x\right)=\frac{1}{1+e^{-2x}}-1$) and sigmoid ($f\left(x\right)=\frac{1}{1+e^{-x}}$) are the most common activation functions used to model a neuron’s output *f* as a function of its input *x*. However, if we take training time with gradient descent into account, these activation functions will be far slower than ReLU [3,25].

In this paper, we use the rectified linear unit to add the non-linearity to our neural network. As shown in (2), when the input is negative, ReLU will output 0. On the other hand, ReLU will return the same value *x* if the input is a positive value:

(2) $$f\left(x\right)=\left\{\begin{array}{c} 0\;\;\;\;\;\;\mathrm{for}\;\;\;x<0,\\ x\;\;\;\;\;\;\;\mathrm{for}\;\;\;x\geq 0 \end{array}\right.$$

### 3.5. Batch Normalization

Batch normalization (BN) is usually used to improve the training performance and enhance the stability of convolutional neural networks [26].

In most neural networks, exploding or vanishing may happen when we use a learning rate that is too large. To address these problems, we apply batch normalization. Normalizing activation across the network helps to avoid small changes happening to the parameters that can lead to amplifying or vanishing these parameters so quickly.

Due to using the batch normalization and ReLU, our algorithm has shown a significant improvement in classification accuracy. In our experiments, batch normalization has contributed to speeding up the training, reducing training and testing time, and lowering the sensitivity to initialization [27].

In our proposed CNN architecture, batch normalization is added before the activation function (ReLU):

(3) y=Wx+b

After normalizing Wx+b, the bias *b* can be canceled and (1) will be:

(4) y=σ(BN(Wx))

In this work, unlike in the fully connected layers, in the convolutional layers, normalization follows the convolution property where at different locations, different elements of the same feature map will be normalized in the same way.

### 3.6. Root Mean Square Propagation Optimizer

Optimization techniques are used to update the network parameters (e.g., weight and bias) in order to generate better results.

Root Mean Square Propagation (RMSprop) optimizer adjusts the learning rate automatically, while the magnitude of the recent gradient is used to normalize the gradient. Consequently, the speed and accuracy of our neural network will be boosted.

The main idea of the RMSprop optimizer is to restrict the oscillations in the vertical direction. Thus, the learning rate will be increased and the algorithm will take larger steps in the horizontal direction rather than vertical direction. Accordingly, training will converge faster and closest to the global minimum point [28,29].

In order to make a good trade-off between low computational cost and high learning rate, we reduce the speed of the learning rate dynamically every *N* epochs when it is necessary. At first, we initialize the learning rate to $10^{-3}$ for all layers, then we divide it by 100 for every N epochs.

As shown in (7) and (8), the learning rate is divided by an exponentially decaying average of squared gradients. In our experiments, we found that the optimal values of decay factor and learning rate are 0.9 and 0.001, respectively. In our proposed model, each update is done according to the following equations, where each parameter is updated individually.

(5) $$Sdw=\beta \cdot Sdw+\left(1-\beta \right)dw^{2}$$

(6) $$S_{db}=\beta \cdot S_{db}+\left(1-\beta \right)dw^{2}$$

After updating the values of weights *W* and bias *b*, the updated weights and biases will be as follows:

(7) $$W=W-\alpha \frac{V_{dw}^{corrected}}{\sqrt{S_{dw}^{corrected}}+\epsilon }$$

(8) $$b=b-\alpha \frac{V_{db}^{corrected}}{\sqrt{S_{db}^{corrected}}+\epsilon }$$

where α is the initial learning rate, *S* is the exponential average of squared gradients, and $V_{db}^{corrected}$ and $V_{dw}^{corrected}$ are the corrected value of db and dw, respectively.

In (5), the exponential average is multiplied by β until the last update, while the square of the current gradient is multiplied by (1−β). Thus, dw corresponds to the gradient component along the direction that is represented by the updated parameter.

### 3.7. Max Pooling

Selecting the maximum value from each region of each feature map is known as the max-pooling operation. Therefore, the result after applying the max-pooling technique will be downsampled feature maps containing the most essential feature of the previous feature map. Thus, we can minimize the number of parameters, reduce the computational complexity, as well as prevent overfitting.

In max pooling, each essential feature is passed to the next layer, while the inessential features are left behind.

In our CNN model, a kernel of the max-pooling layer of 2 × 2 with a stride of 2 is applied along the spatial dimensions of the input.

**Figure 7:**
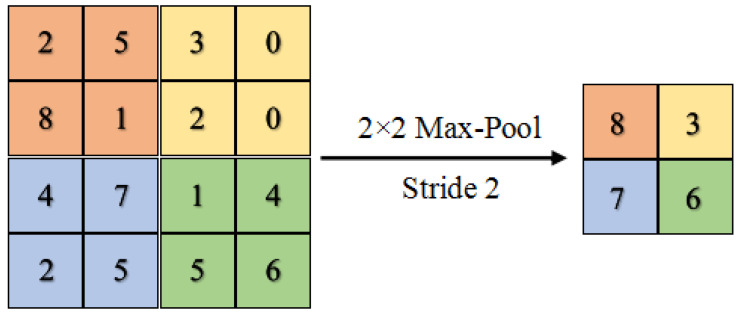
Maximum pooling with filter size 2 × 2 and stride of 2.

As shown in Figure 7, we extract the patches from input feature maps, take the maximum value of each patch as output, and disregard the rest of the values. Unlike height and width of the feature maps, we leave the depth dimension without changing it (as illustrated in Figure 6) [30]. In other words, when the size of the input is $W_{in}\times H_{in}\times D$, the size of the output will be $W_{out}\times H_{out}\times D$, where:

(9) $$W_{out}=\frac{W_{in}-F}{S}+1$$

(10) $$H_{out}=\frac{H_{in}-F}{S}+1$$

where *S* and *F* refer to stride and filter size, respectively. From these two formulas, we observe that the width and height of the feature map have changed, while the depth has been kept unchanged.

The combination of convolutional layers and pooling layer enables our model to combine extracting local features and learning further global features of the input image.

### 3.8. Dropout

Dropout is a regularization technique that randomly removes some selected neurons during training. Removing the selected neurons usually results in less sensitivity to particular weights. Furthermore, randomly dropping neurons helps to force the neural network to learn features distributedly. Dropout is usually used in the large network to improve the generalization and tackle the overfitting problem that may lead to complex co-adaptations on the training data [31,32]. In addition to that, dropout makes the neural network more sturdy and less dependent on specific idiosyncrasies of the training data. Applying dropout to all hidden and visible layers usually achieves better results than applying it to only one hidden layer [32]. Thus, in our experiments, neurons in feature extraction layers are randomly removed from the neural network with a probability of 0.25, and 0.5 in classification layer.

### 3.9. Fully Connected Layer

In the image classification task, the fully connected layer comes as the last layer, where the neurons in the fully connected layer are linked to all neurons of the preceding layers to combine all local features of these layers.

In the fully connected layer, feature maps learned from previous convolutional layers are collected to provide us with the potential classification score of the image in the input layer. In this layer, the feature maps are converted into a one-dimensional vector.

Based on training data, in our fully connected layer, softmax is used as an activation function to classify the learned features collected from preceding layers into different classes and show the probabilities of each class.

### 3.10. Softmax Function

In the multi-class classification neural network, relative magnitudes of all neuron outputs should be taken into account. For that reason, softmax function is the perfect choice for multi-class classification neural networks [33]. In the softmax function, the total of the output values is one, while each individual output value is between 0 and 1. The mathematical formulation of the softmax activation function can be expressed as follows:

(11) $$\theta {\left(z\right)}_{i}=\frac{e^{z_{i}}}{\sum_{k=1}^{M}e^{z_{k}}}$$

where $z_{i}$ refers to the weighted sum of the *i*-th output neuron, while *M* is the number of output neurons.

In this work, softmax activation function classifies the final outputs into ten categories of MNIST dataset (numbers from 0 to 9), where each category corresponds to a specific probability distribution.

### 3.11. Data Augmentation

Data augmentation creates new training data from existing training data and adds this data to the original dataset to prevent overfitting. To avoid the overfitting problem, we change the training data by modifying the array representation and keep the label without change.

With the aim of continuing to decrease the validation errors with the training errors, we utilize a data augmentation technique as a super-powerful technique of achieving this goal. By applying data augmentation, a comprehensive set of possible data points will be represented which will contribute to minimizing the difference between the training and the validation sets [24].

In this paper, we use random rotation, zoom range, random horizontal shift image, and random vertical shift image. Because of the neuter of the handwritten numbers, we avoid applying horizontal flipping and vertical flipping. Applying horizontal/vertical flipping can cause misrecognition of symmetric numbers like 9 and 6.

#### 3.11.1. Flipping

Flipping is easy to implement, and the horizontal axis flipping is used more than the vertical one. Flipping is not suitable for datasets that contain text such as an MNIST handwritten dataset. Therefore, we avoid using data augmentation with flipping in our experiments.

#### 3.11.2. Rotation

Image rotation is common in image processing, where the image rotates left or right by a given number of degrees from 0 to 360. Rotation augmentation is determined by the rotation degree parameter. Slight rotations (5–20 degrees) have a significant positive effect on handwritten digit recognition such as in MNIST datasets. On the other hand, the data label will lose the post-transformation as the degree of rotation increases. Moreover, in the case of handwritten digits, rotated digits seem to have different meanings; for example, when digit 6 rotates 180 degrees, it will look like digit 9 [34]. In our experiments, images are randomly rotated in a range of 10 degrees.

#### 3.11.3. Shifting

Left-, right-, up-, or down-shifting is very helpful to avert data positional bias. In the case that all images in a dataset are centered, testing on perfectly centered images is required [24].

When we shift the entire image horizontally or vertically, all image pixels will move in one direction, while image dimensions will remain the same. Translating an image in a direction will leave a space which should be filled with some constant values such as zero or 255, otherwise this space will be filed with the noise.

In this work, the random vertical and horizontal shifts of the data training have been set to 0.1.

### 3.12. Loss Function

In this subsection, we use the loss function to measure how great our model performs in regard to predicting the expected output digit that has a known label.

Categorical cross-entropy is more suitable for multiclass classification missions, especially MNIST handwritten digit classification. In our CNN architecture, categorical cross-entropy is used to learn to give more likelihood to the correct digit and less likelihood to the other digits.

There is an inverse relationship between the cross-entropy loss and the predictive likelihood, where the cross-entropy loss increases as the predictive likelihood diverges from the correct label.

The formulation we use to calculate the cross-entropy loss function can be implemented as follows:

(12) $$Loss=-\sum_{c=1}^{M}y_{i}\cdot \log\;{\hat{y}}_{i}$$

where *M* is the number of classes, $\hat{y}$ is predicted value, and *y* is the actual value.

### 3.13. Receptive Field

Receptive field is a defined region on the output volume of the preceding layer that provides input to a set of neurons within the next layer.

**Figure 8:**
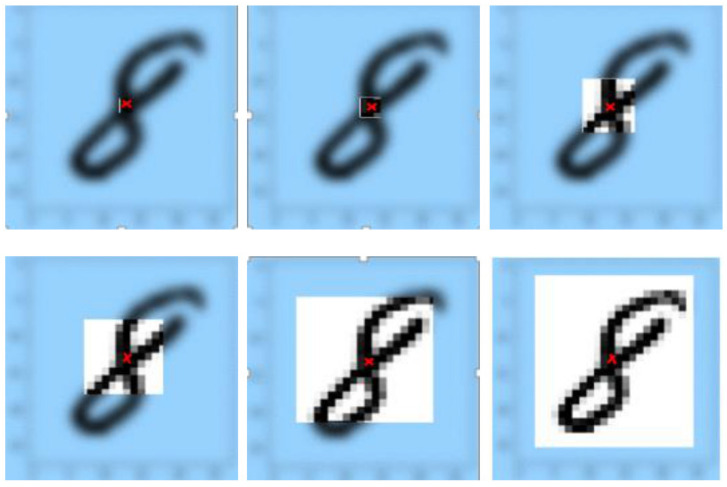
ERF of neurons in different layers. From left- to right-hand side and from top to bottom: images with ERF size of: 3 × 3, 4×4, 8×8, 10×10, 18×18, and 22×22.

Figure 8 shows the influence of receptive fields across the layers, for which the input image is a 28 × 28 matrix. A 3 × 3 filter is used to convolve the image to gain its feature map. Then, the process is repeated with the next layers to gain deeper feature maps until we get the output image with an effective receptive field with a size of 22 × 22.

In the receptive field, pixels have different contributions to the output layer response. In forward propagation, the central pixels of the receptive field propagate the image’s information to the output across multiple different paths. In contrast, outer pixels propagate the information only across a small number of paths, and that is why the outer area of the receptive field has only very limited influence [35,36]. In backpropagation, since central pixels propagate the gradients in the output layer through all paths, the central pixels will have a wider magnitude for the gradient than that of the outer pixels.

### 3.14. ERF Calculation

The effective receptive field (ERF) refers to the areas of the input image that should be taken into consideration during the filtering process. Hence, neuronal activity will be modified according to the range of the input image.

To calculate ERF of a neuron at layer *i*, let us suppose that *R* is ERF at the current layer (*i*) and $R_{i-1}$ is ERF of a neuron at the preceding layer (i−1). To start the calculation of ERF, we add a non-overlapped-area *N* to $R_{i-1}$:

(13) $$R_{i}=R_{i-1}+N$$

Now, let us refer to the filter size in layer *i* by $f_{i}$. Then, the number of the filters that are overlapping with each other will be $f_{i}-1$.

As is well known, convolving the filter with a stride that is larger than one can cause a great increase in the non-overlapped area. For that reason, accounting for the number of pixels that each extra filter has contributed to ERF is extremely needed.

Due to the effect of the lower layer stride on the ERF of the higher layer, accumulating the pixel contributions in all layers is highly necessary. Accordingly, the non-overlapped area is calculated as follows:

(14) $$N=\left(f_{i}-1\right)\prod_{k=1}^{i-1}S_{k}$$

where $S_{k}$ refers to the stride in the layer *k*.

By combining (13) and (14) we can obtain the general formula that is used to compute ERF [36]:

(15) $$R_{i}=R_{i-1}+\left(f_{i}-1\right)\prod_{k=1}^{i-1}S_{k}$$

In each layer, the calculation of the size of the output feature can be expressed as follows:

(16) $$n_{i}=\left[\frac{n_{i-1}+2p-k}{S}\right]+1$$

In output feature map, we can calculate the distance between two adjacent features (jump) by the following formula:

(17) $$j_{i}=j_{i-1}*S$$

The following formula calculates the center position of the receptive field:

(18) $$C_{i}=C_{i-1}+\left(\frac{k-1}{2}-p\right)*j_{i-1}$$

where *p* is the padding size and *k* is the filter size.

The results of calculating jump, center position, and ERF can be found in Table 2 and Figure 8.

## 4. Experimental Results

The description of this section is organized as follows: the Experimental Setup, Neural Network Architecture, Early Stopping, and Quantitative Metrics and Visual Quality are described in Section 4.1, Section 4.2, Section 4.3 and Section 4.4, respectively. Comparisons with State-of-the-Art Models, Results with/without Data Augmentation, and Noisy MNIST Dataset are presented in Section 4.5, Section 4.6 and Section 4.7, respectively.

### 4.1. Experimental Setup

As an essential first step for our data preparation process, the mean, standard deviation, and feature density of training and testing sets were calculated and plotted before applying our CNN model. Plotting data can allow us to determine if variables are related, and how much they are related. Thus, we can define if there are any redundant variables or irrelevant variables to the target variable.

In the real world, images can be exposed to natural influences that can affect image quality which makes them hard to recognize. In this work, additive white Gaussian noise with σ=0.5 is added to the MNIST dataset to simulate these natural influences. Then, we evaluate the classification performance of our model on a noisy MNIST dataset to verify the classification ability of this model in a case dealing with high levels of noise.

In this work, filter sizes are determined by calculating the size of the ERF. Calculating this size can help in enhancing the performance of our CNNs. In our proposed algorithm, the receptive field is designed so that it can cover the whole relevant input image area. In our experiments, we increase our receptive field size by utilizing max-pooling with a 2 × 2 filter and stride = 2.

Experimental results show that our proposed model has a robust image classification performance even with massive noise levels.

The comparison with the remaining models shows that the accuracy of our proposed model is very competitive with the best results achieved by other models.

### 4.2. Neural Network Architecture

Our experimental procedure includes feature extraction and classification of the handwritten digit images. With the aim of capturing enough spatial information for classification, eight different types of neural network architectures with different neurons and layers are utilized. In this work, all convolutional layers have 3×3 filters. Nevertheless, these layers differ from each other in the number of filters. The downsampling of our convolutional layers is performed in many different ways, such as max-pooling with a stride of 2, and dropout. In the hidden layers, we use dropout with probability *p* = 0.25, and *p* = 0.5 in the fully connected layer. The reason for using a small dropout rate in the hidden layers is because the typical value p=0.5 may lead to slower convergence and higher error rates. In our CNN, there are three hidden layers, a fully connected layer, and an output layer. For the three hidden layers, 32, 26, and 128 filters of size 3×3 are used to produce 26, 11, and 3 feature maps, respectively. The combination of convolutional, max pooling and batch normalization layers enables our network to extract abundant local features combined with learning more global features of the input image. In our model, we utilize RMSprop instead of SGD. RMSprop is not only restricted to sum of the past gradient but also restricted to gradients for the recent time steps. For this reason, our model converges faster than the one that uses traditional SGD.

To improve the stability of our model, we use epsilon = 1 × 10^−8^, roh=0.9, and a learning rate of 0.001.

### 4.3. Early Stopping

During the process of training dataset, overfitting or underfitting happens when the model respectively has a large or small number of epochs. The early stopping technique is a powerful and effective way to prevent overfitting and underfitting in ConvNet models. In the early stopping technique, we can determine a specific number of training epochs and stop the training process once the accuracy starts getting worse or even stops improving. In our experiments, we found applying early stopping has an enormously positive impact on classification accuracy.

### 4.4. Quantitative Metrics and Visual Quality

**Figure 9:**
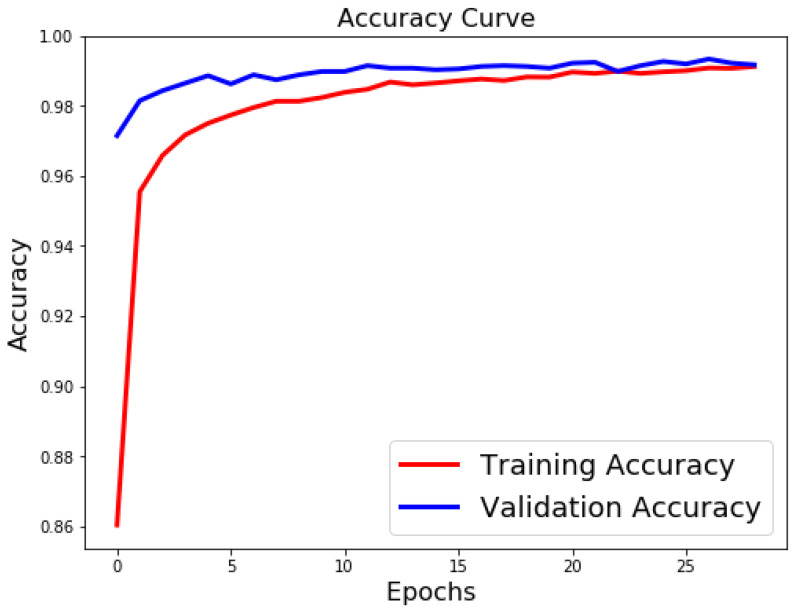
Classification accuracy for case 1 (without data augmentation).

**Figure 10:**
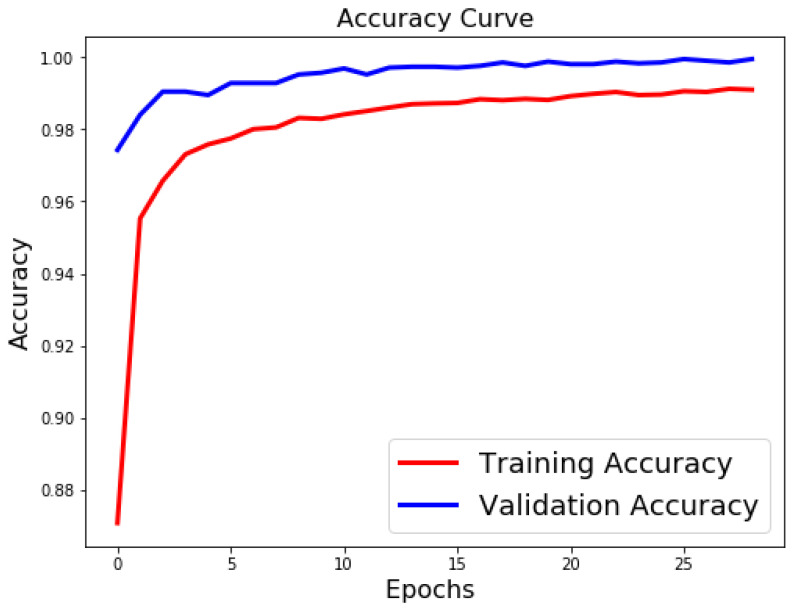
Classification accuracy for case 1 (with data augmentation).

**Figure 11:**
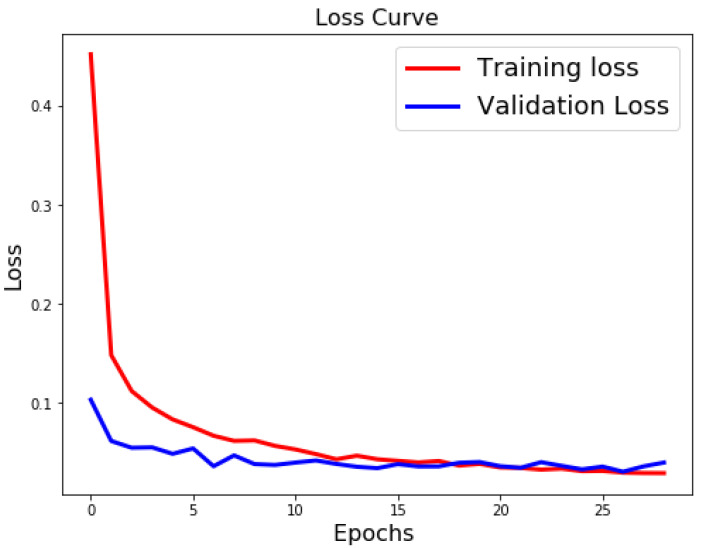
Classification loss for case 1 (without data augmentation).

**Figure 12:**
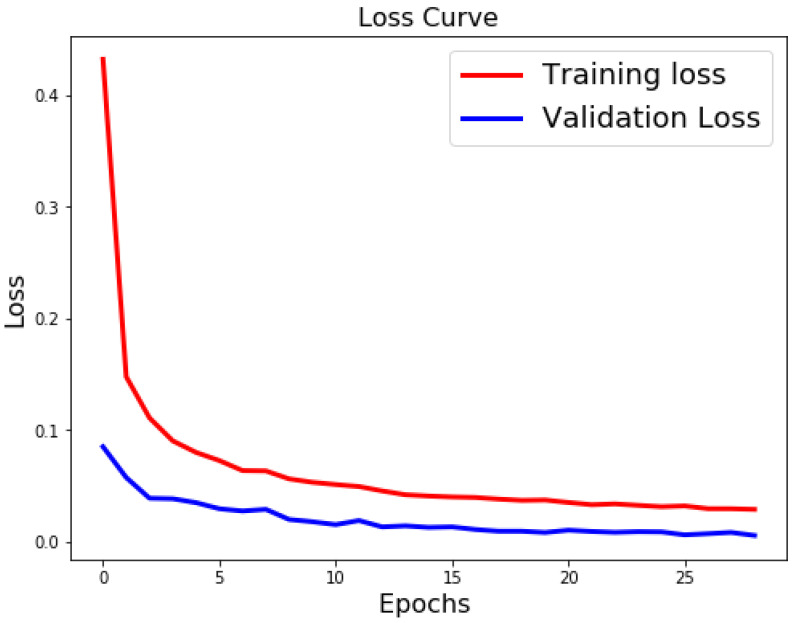
Classification loss for case 1 (with data augmentation).

**Figure 13:**
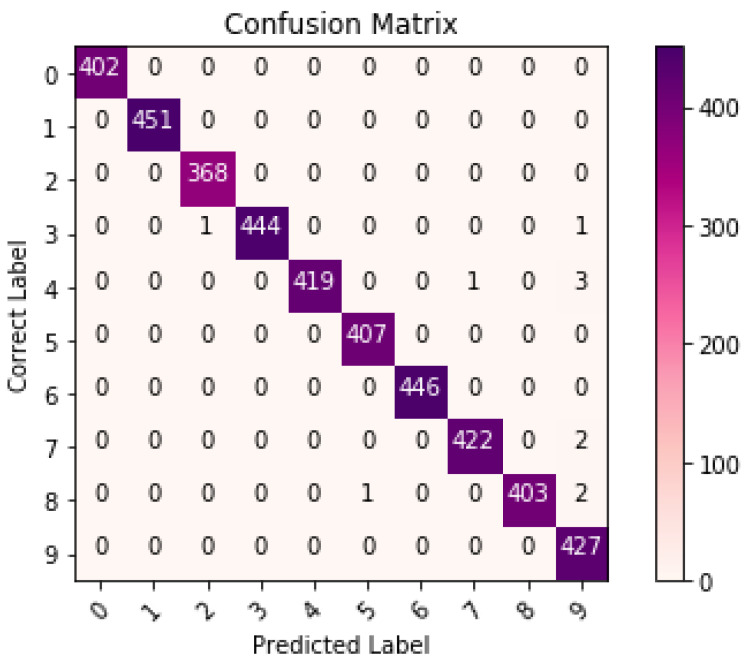
Confusion matrix graph of our proposed CNN.

**Figure 14:**
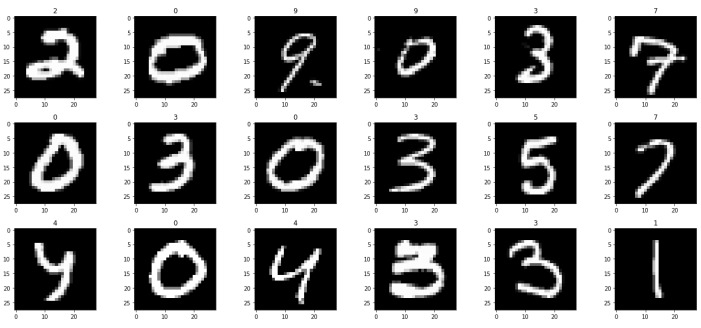
Sample MNIST handwritten digit images predicted by the proposed CNN model.

Table 3, Table 4 and Table 5 list the results of 4-layer, 3-layer, and 5-layer convolutional neural networks with the implementation of data augmentation, where the maximum epoch counts of 29. These tables include the results of training loss, validation loss, training accuracy, validation accuracy, and running time for cases 1 to 8. In these tables, the highest accuracy achieved for handwritten digit recognition is 99.98% (Table 5). In contrast, Table 6, Table 7 and Table 8 show the results of 4-layer, 3-layer, and 5-layer convolutional neural networks without the implementation of data augmentation, and maximum epoch counts of 29. The highest accuracy achieved in these tables is 99.50% (Table 7 and Table 8). Table 3, Table 4, Table 5, Table 6, Table 7 and Table 8 show that the convolutional neural networks with data augmentation have achieved higher validation (testing) and training accuracies than convolutional neural networks without data augmentation. To discover the reasons, we compare their validation and training losses during the validation and training procedures. From Table 3, Table 4, Table 5, Table 6, Table 7 and Table 8, we can discover that convolutional neural networks (without data augmentation) in Table 6, Table 7 and Table 8, have higher validation and training lossesthan thatin Table 3, Table 4 and Table 5. In Figure 9 and Figure 10, we visualize the positive correlation between accuracy and the number of epochs. Figure 9 and Figure 10 show the validation and training accuracies with and without data augmentation, respectively. From Figure 10, we can observe that applying data augmentation can result in faster and more stable convergence than that in Figure 9. This is due to the fact that utilizing data augmentation results in an increase in the diversity of data available for training. Consequently, our model can learn more from a wide range of features. All this reflects on improving the accuracy of our model. Data augmentation can also speed up the training and enhance the performance. From Figure 10, we can notice that at the very beginning, the curve of validation accuracy suffered from jitter. Nevertheless, at epoch 12, the curve started to converge slowly until it became more smooth at the end of the curve. This stability without any obvious performance drop is a result of utilizing data augmentation technique. In contrast, in Figure 9, the curve of validation accuracy still jitters heavily even after epoch 12. By comparing Figure 11 to Figure 12, we can discover that utilizing data augmentation in our model helps to stabilize training and validation processes without any sudden rise in the validation loss curve. On the other hand, as shown in Figure 11, convolutional neural network without the implementation of data augmentation shows a significant drop and rise in the validation loss curve, especially at both ends of the curve. Figure 13 shows the confusion matrix of our proposed neural network. In this figure, we use a confusion matrix to evaluate the performance of our classification model. From this figure, we can notice that more than 99% of the values (diagonal elements) that represent the number of points in which the true label is equal to the predicted label lie on the main diagonal. In contrast, only less than 1% of the values (off-diagonal elements) that represent the mislabeled elements lie outside the main diagonal. Some of the MNIST handwritten digit images predicted by the proposed CNN technique are presented in Figure 14.

As shown in Figure 8 and Table 2, the ERF sizes are 3 × 3, 4 × 4, 8 × 8, 10 × 10, 18 × 18, and 22 × 22 pixels, while the center positions are 1.5, 2, 4, 7, 10, and 13. As we can see, the last size (22 × 22) of ERFcovers the entire image.

In our experiments, we increase our receptive field size by utilizing max-pooling with a 2 × 2 filter and stride = 2.

### 4.5. Comparisons with State-of-the-Art Models

In Table 9, we compare our proposed model with the recent handwritten digit classification methods. It is obvious to see that our proposed model yields the highest classification accuracy in all cases. Our proposed model outperforms the competing models by 0.1 to 5.6.

The results of the reference methods are cited from their papers.

The results listed in Table 9 demonstrate that our model holds the overwhelming superiority over state-of-the-art models in terms of classification accuracy. From this table, we can observe that our model has achieved a classification accuracy of 99.98%.

### 4.6. Results with/without Data Augmentation

**Figure 15:**
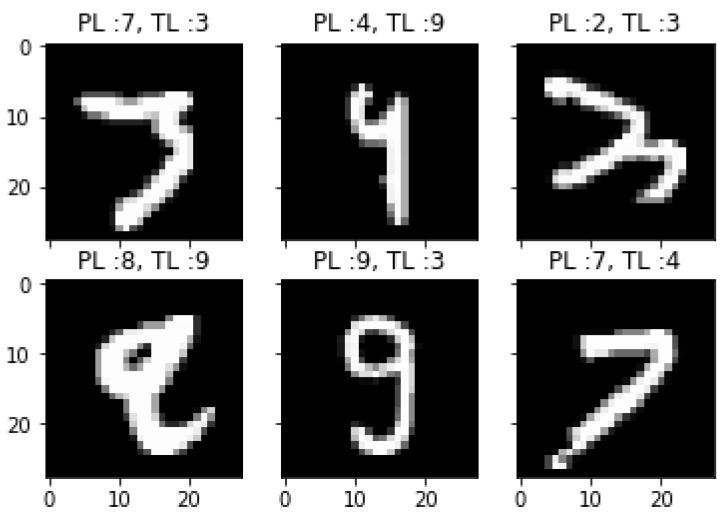
Some error results predicted by CNN without data augmentation (where PL is predicted label, TL is true label).

The core idea behind applying data augmentation is to avert the overfitting problem by artificially increasing the size of our datasets without collecting new data. We can do that by making tiny transformations to the existing training data, while keeping the labels without change. These small transformations act as a regularizer, which assists us in reducing overfitting. In our experiments, we randomly rotate the images in a range of 10 degrees. Zoom range, random vertical, and horizontal shifts of the data training have been set to 0.1. The highest accuracy that was achieved with data augmentation was 99.98%, and only 99.50% without data augmentation. Figure 15 shows some labeling error results predicted by convolutional neural network without adopting data augmentation. As we can see, the numbers 3, 4, and 9 are misclassified as 2, 7, and 8 respectively. The main reason for this misclassification may be that the smooth curves of these numbers make them hard to classify. From this figure, it is clear to see that digit 3 is completely misleading and looks very similar to 9.

### 4.7. Noisy MNIST Dataset

Images in the real world can be exposed to some of the natural influences such as dust, air pollution, and a number of other abnormalities. In order to simulate the performance of our proposed algorithm for these natural influences, we add an additive white Gaussian noise with σ=0.5 to the MNIST dataset.

In this subsection, we evaluate the classification performance of our proposed algorithm on a noisy MNIST dataset to verify its classification ability in a case dealing with high levels of noise.

In our experiments, all images in the training and testing sets are corrupted with (σ=0.5) random noise (additive white Gaussian noise). As shown in Table 10, our proposed model performs well even with massive noise levels, which can achieve an amazing classification accuracy of up to 99.40%.

**Figure 16:**
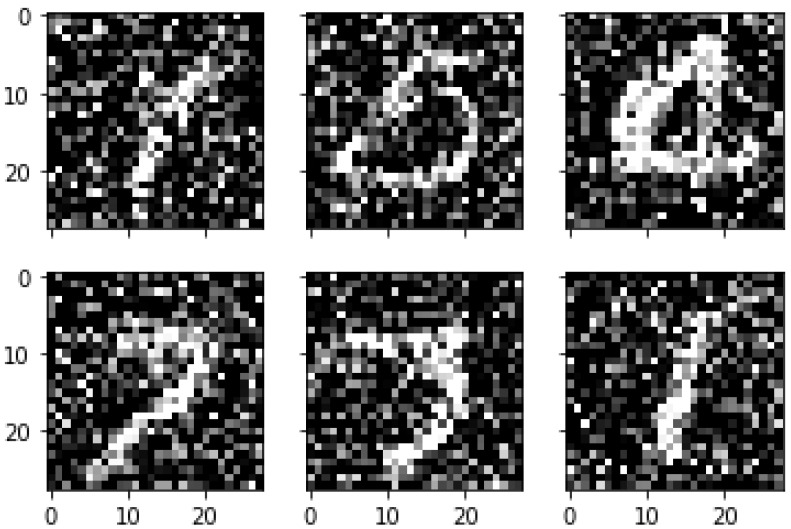
Sample of MNIST handwritten digit images corrupted with 50% noise.

Figure 16 shows some of the MNIST handwritten digit images obtained by adding additive white Gaussian noise (σ=0.5). From Figure 16, we can see that the images have been seriously damaged by a massive amount of additive white Gaussian noise, which makes the classification mission of these images extremely hard even for the human eye. Nevertheless, our proposed algorithm has achieved great classification results with a recognition accuracy of 99.40%.

Table 10 lists the results of training loss, validation loss, training accuracy, validation accuracy, and running time. The results listed in this table have been achieved by the proposed convolutional neural network after adding 50% noise. In this table, the highest accuracy achieved for the noisy handwritten digit recognition is 99.40%.

In Table 11, we compare our proposed model with the different handwritten digit classification models. From Table 11, we can easily observe that our proposed algorithm gains the highest recognition accuracy compared with other algorithms.

In this table, the results of the reference algorithms have been cited from their papers. As shown in this table, our proposed algorithm surpasses the competing algorithms by 0.9 to 5.18.

To highlight the advantages of the robustness of the proposed algorithm, we compare the performance of our proposed algorithm with other existing algorithms for noisy MNIST dataset classification over the same noise level (σ=50). Experimental results demonstrated that our proposed algorithm not only offers pleasing results but also surpasses all of the other reference algorithms in terms of noisy image classification. Based on Table 11, we can observe that our proposed model is very effective in regard to classifying massive noisy images (50% noise). In this table, our proposed algorithm has achieved significant improvements over the algorithms in [48,49], with 3.06% and 2.99% gains higher than [48,49], respectively. Adopting effective receptive field (ERF), data preparation, and data augmentation in our proposed algorithm led to significant enhancements in the classification accuracy as compared with that in the existing state-of-the-art algorithms.

## 5. Discussion

In the following subsections, we offer a brief overview of advantages, limitations, and the future of our proposed model.

### 5.1. Advantages of Proposed Model

In our proposed model, the data augmentation technique serves as the essential build module. Our model takes the advantage of applying data augmentation to modify the original limited dataset (MNIST dataset) with a view towards possessing big data characteristics. In this sense, extracting extra information from the original dataset enables us to obtain training data with improved quality and size, which helps in preventing overfitting in our neural network.

Inspired by the fact that a large number of epochs may result in overfitting, and a small number of epochs usually leads to underfitting, our model adopts the early stopping technique to determine the optimal number of training epochs.

In order to correctly initialize the dataset to be fit for utilizing in our proposed CNN model, data preparation is conducted as an essential first step of our proposed model. By applying the data preparation process, we can determine whether there are any redundant variables or irrelevant variables to the target variable.

To simulate the real-world natural influences that can affect image quality, which in turn influences the classification accuracy of the image, additive white Gaussian noise with σ=0.5 was added to the MNIST dataset. Then, the noisy MNIST dataset is used to evaluate the classification performance of our proposed algorithm.

Selecting the perfect filter of the appropriate size is based on calculating the size of ERF and domain knowledge, while the receptive field is designed to be able to cover the whole relevant input image area. All these factors have led to great enhancements in image classification accuracy in our model.

### 5.2. Limitations of Our Model

As we can see in Figure 13, in some cases there is an amount of almost 1% of the values that have exist outside the main diagonal, which means that our proposed algorithm still produces some of the mislabeled elements. The principal reason for the failure of our model to correctly labeled these elements could be that the different handwritten digit images vary in size and style, which makes the classification mission much harder even for humans. In addition to that, our CNN model with data augmentation consumes a bit more time than CNN without data augmentation because of the extra data that has been extracted from the original dataset.

### 5.3. Future Works

We believe that our proposed model can further be applied to other datasets. In contrast, as a future work, we find that it is worth taking further actions to improve our model performance in terms of how to perfectly learn and extract the local features in the hidden layers, and how to enhance the recognition ability in the fully connected layers to avoid mislabeling problems.

## 6. Conclusions

In this paper, we presented a novel convolutional neural network architecture based on data preparation, receptive field, data augmentation, optimization, normalization, and regularization techniques for handwritten digit recognition. To guarantee the dataset does not contain any unnecessary details and that it is fit for applying in our CNN model, data preparation is conducted as an essential first step in our proposed model. Without applying data preparation to the raw data, it is highly possible that unnecessary data leads to misleading results. In our work, filter sizes are determined by calculating the size of the ERF. Calculating this size can help in enhancing the performance of our CNN. In ERF, the process is started with a proposed filter to convolve the input image and gain its feature map. Then, this process is repeated with the next layers to gain deeper feature maps until getting the output image with an effective receptive field with a size of 22 × 22. Max-pooling with a 2 × 2 filter and stride = 2 is used to increase the size of the receptive field. Proposed CNN architecture has achieved recognition accuracy of 99.98% on the MNIST handwritten digit dataset, and 99.40% with the same dataset contaminated with 50% noise. Our experimental results show that using data augmentation with CNN gives better recognition accuracy compared to CNN without data augmentation. Utilizing the data augmentation technique has helped to expand our training dataset, resulting in improving the performance and classification capability of our model. We also presented the RMSprop optimizer to restrict the oscillations in the vertical direction and take larger steps in the horizontal direction in order to converge substantially faster to the global minimum point. On the very competitive MNIST handwritten digits benchmark, our proposed CNN model has achieved superiority over state-of-the-art methods for handwritten digits recognition. In our experiments, batch normalization has been used to improve the training performance and enhance the stability of our model. With the usage of batch normalization, we can speed up the training, reduce training and testing time, in addition to lowering the sensitivity initialization. In order to avoid overfitting and underfitting, an early stopping technique has used to determine the optimal number of training epochs.

## Figures and Tables

**Table 1:** Distribution of label values in the training set.

Label	0	1	2	3	4	5	6	7	8	9
Count	4132	4684	4177	4351	4072	3795	4137	4401	4063	4188

**Table 2:** ERF size, jump, and the center position of the receptive field for different layers.

Layer	Colv 1	Pool 1	Conv 2	Pool 2	Conv 3	Pool 3
ERF	3	4	8	10	18	22
Jump	1	2	2	4	4	8
C. P.	1.5	2	4	7	11	13

**Table 3:** Loss and accuracy achieved by 4-layer convolutional neural network (with data augmentation).

Case	Epoch	T.loss	Val.loss	T.acc	Val.acc	Time
Case.1	1	0.4323	0.0849	0.8708	0.9743	66 s
Case.1	15	0.0407	0.0125	0.9872	0.9974	66 s
Case.1	29	0.0287	0.0053	0.9910	0.9995	61 s
Case.2	1	0.3638	0.1201	0.8864	0.9686	126 s
Case.2	15	0.0194	0.0074	0.9942	0.9988	113 s
Case.2	29	0.0129	0.0044	0.9963	0.9995	113 s
Case.3	1	0.3672	0.0616	0.8810	0.9810	28 s
Case.3	15	0.0241	0.0066	0.9922	0.9990	29 s
Case.3	29	0.0135	0.0043	0.9955	0.9988	27 s
Case.7	1	0.1748	0.0549	0.9443	0.9838	218 s
Case.7	15	0.0129	0.0076	0.9958	0.9983	216 s
Case.7	29	0.0091	0.0046	0.9974	0.9995	216 s

**Table 4:** Loss and accuracy achieved by 3-layer convolutional neural network (with data augmentation).

Case	Epoch	T.loss	Val.loss	T.acc	Val.acc	Time
Case.5	1	0.4404	0.0644	0.8628	0.9824	56 s
Case.5	15	0.0299	0.0160	0.9914	0.9974	55 s
Case.5	29	0.0229	0.0082	0.9931	0.9981	56 s
Case.6	1	0.2043	0.0498	0.9350	0.9855	52 s
Case.6	15	0.0118	0.0076	0.9963	0.9988	50 s
Case.6	29	0.0061	0.0049	0.9980	0. 0.9995	49 s
Case.8	1	0.2424	0.0444	0.9220	0.9862	225 s
Case.8	15	0.0228	0.0099	0.9937	0.9979	224 s
Case.8	29	0.0269	0.0090	0.9931	0.9974	230 s

**Table 5:** Loss and accuracy achieved by 5-layer convolutional neural network (with data augmentation).

Case	Epoch	T.loss	Val.loss	T.acc	Val.acc	Time
Case.4	1	0.3152	0.0511	0.8960	0.9855	55 s
Case.4	15	0.0060	0.0089	0.9984	0.9976	54 s
Case.4	29	0.0051	0.0039	0.9990	0.9998	53 s

**Table 6:** Loss and accuracy achieved by 4-layer convolutional neural network (without data augmentation).

Case	Epoch	T.loss	Val.loss	T.acc	Val.acc	Time
Case.1	1	0.4520	0.1029	0.8603	0.9714	80 s
Case.1	15	0.0427	0.0337	0.9865	0.9902	80 s
Case.1	29	0.0287	0.0394	0.9912	0.9917	77 s
Case.2	1	0.3688	0.0696	0.8845	0.9802	80 s
Case.2	15	0.0214	0.0264	0.9933	0.9940	79 s
Case.2	29	0.0120	0.0232	0.9959	0.9955	81 s
Case.3	1	0.3812	0.0742	0.8762	0.9774	36 s
Case.3	15	0.0244	0.0347	0.9918	0.9919	35 s
Case.3	29	0.0155	0.0285	0.9949	0.9952	34 s
Case.7	1	0.1870	0.0603	0.9413	0.9826	167 s
Case.7	15	0.0130	0.0380	0.9953	0.9895	167 s
Case.7	29	0.0101	0.0315	0.9967	0.9948	166 s

**Table 7:** Loss and accuracy achieved by 3-layer convolutional neural network (without data augmentation).

Case	Epoch	T.loss	Val.loss	T.acc	Val.acc	Time
Case.5	1	0.4734	0.0763	0.8535	0.9805	50 s
Case.5	15	0.0273	0.03370	0.9920	0.9924	49 s
Case.5	29	0.0156	0.0360	0.9952	0.9936	49 s
Case.6	1	0.2211	0.0577	0.9304	0.9812	45 s
Case.6	15	0.0114	0.0400	0.9960	0.9924	44 s
Case.6	29	0.0050	0.0527	0.9983	0. 0.9924	44 s
Case.8	1	0.2724	0.0587	0.9129	0.9814	208 s
Case.8	15	0.0201	0.0293	0.9944	0.9950	199 s
Case.8	29	0.0198	0.0333	0.9950	0.9938	199 s

**Table 8:** Loss and accuracy achieved by 5-layer convolutional neural network (without data augmentation).

Case	Epoch	T.loss	Val.loss	T.acc	Val.acc	Time
Case.4	1	0.3618	0.1263	0.8829	0.9576	37 s
Case.4	15	0.0076	0.0347	0.9980	0.9950	37 s
Case.4	29	0.0054	0.0726	0.9989	0.9921	35 s

**Table 9:** Quantitative results of different models on MNIST dataset.

Algorithm	Approach	Database	Accuracy (%)
Ref. [37]	CNN	MNIST	99.00%
Ref. [9]	CNN	MNIST	99.21%
Ref. [38]	CNN	MNIST	99.89%
Ref. [39]	CNN	MNIST	98.10%
Ref. [40]	CNN	MNIST	97.50%
Ref. [41]	CNN	MNIST	98.00%
Ref. [10]	CNN	MNIST	98.00%
Ref. [12]	DCST	MNIST	98.80%
Ref. [42]	CNN	MNIST	98.45%
Ref. [13]	LIRA	MNIST	99.41%
Ref. [20]	SNN	MNIST	98.17%
Ref. [43]	ASSOM	Handwritten digits	99.30%
Ref. [14]	CNN/SVM	MNIST	99.28%
Ref. [17]	OPF	MNIST	99.35%
Ref. [44]	DM	NIST SD-1	94.70%
Ref. [22]	SNN/SVM	MNIST	94.40%
Ours	CNN	MNIST	99.98%

**Table 10:** Loss and accuracy achieved by the proposed convolutional neural network (with adding 50% noise).

Epoch	T.loss	Val.loss	T.acc	Val.acc	Time
1	0.9629	0.3506	0.7074	0.8929	81 s
15	0.1305	0.0387	0.9575	0.9886	79 s
29	0.0975	0.0192	0.9688	0.9940	79 s

**Table 11:** Quantitative results of different models on noisy MNIST dataset.

Algorithm	Approach	Noise	Accuracy (%)
Ref. [45]	SRNN	25%	98.43%
Ref. [46]	Sda-3	10%	97.20%
Ref. [47]	SVM	25%	98.37%
Ref. [47]	SDAE-3	25%	98.50%
Ref. [48]	DAE-CNN	50%	95.01%
Ref. [48]	CDAE-CNN	50%	94.22%
Ref. [48]	DAE-CDAE-CNN	50%	96.34%
Ref. [49]	CNN	20%	98.56%
Ref. [49]	CNN	50%	96.41%
Ours	CNN	50%	99.40%

## Data Availability

The MNIST handwritten digit database can be found in the link below: http://yann.lecun.com/exdb/mnist/ (accessed on 14 September 2021).

## Abbreviations

The following abbreviations are used in this manuscript:

MDPI	Multidisciplinary Digital Publishing Institute
CNN	Convolutional Neural Network
ERF	Effective Receptive Field
MNIST	Modified National Institute of Standards and Technology
ReLU	Rectified Linear Unit
BN	Batch Normalization
RMSprop	Root Mean Square Propagation
